# An Experimental Study of Dynamic Compression Performance of Self-Compacting Concrete

**DOI:** 10.3390/ma13173731

**Published:** 2020-08-24

**Authors:** Feiting Shi, Peng Cao, Ziyu Wang, Yanan Gan, Changjun Zhou, Ketong Liu

**Affiliations:** 1 Civil Engineering Department, Yancheng Institute of Technology, Yancheng 224051, China; shifeiting@ycit.cn (F.S.); gyn_12@126.com (Y.G.); 2 College of Architecture and Civil Engineering, Beijing University of Technology, Beijing 100124, China; 3 School of Ecological Environment, Hainan Tropical Ocean University, Sanya 572022, China; zywang@hntou.edu.cn; 4 School of Transportation and Logistics, Dalian University of Technology, Dalian 116024, China; zhouchangjun@dlut.edu.cn; 5 College of Architecture and Civil Engineering, Xi’an University of Science and Technology, Xi’an 710054, China; ketong-1982@163.com

**Keywords:** self-compacting concrete, uniaxial compression, dynamic properties, experimental study, mechanism analysis

## Abstract

To investigate the dynamic performance of self-compacting concrete (SCC), the dynamic uniaxial compression tests at eight different loading strain rates were performed on the ordinary concrete and SCC cubic specimens. Based on the tests, the compression failure patterns and stress–strain curves of both kinds of concrete were obtained. The results show that SCC performs more brittle than ordinary concrete by showing the diagonal crack failure pattern of SCC at a high strain rate. Besides, with the increase of loading strain rate, the peak compressive stress of SCC is slightly lower than that of ordinary concrete, but the increase of elastic modulus is slightly higher than that of ordinary concrete. The peak compressive strains of the two kinds of concrete are discrete under the influence of loading strain rate, thus putting forward the relation equation for the loading strain rate and peak compressive stress increase coefficient of the two kinds of concrete. Besides, based on the theory of elastic–plastic damage and considering the dynamic extension of damage, the dynamic constitutive relation with good applicability between ordinary concrete and SCC was established.

## 1. Introduction

Self-compacting concrete (SCC) is a kind of high-performance concrete compacted and formed by its weight without compaction. With advantages such as high homogeneity, high compactness, and simplification of construction, it has been widely used and popularized [1,2,3]. Under the service life of a concrete structure, it is not only subjected to static load but also may be subjected to dynamic load. Therefore, it is a need to study the dynamic behavior of concrete [4,5].

To evaluate the dynamic properties of concrete, compression and tension tests are commonly used. Sparks [6] and Bischoff [7] are the pioneers to study the effect of dynamic strain rate on the dynamic performance of concrete and concluded that the stress–strain curve of concrete was significantly affected by the dynamic load rate. Li investigated the dynamic performance of concrete by a large number of experiments and analyzed the failure pattern of concrete based on the theory of elastic–plastic damage [8,9]; Song further studied the effect of large aggregate and wet screened concrete on the dynamic performance of concrete [10,11]. Those related studies found that with the increase of loading strain rate, the strength and elastic modulus of concrete gradually increase, and the variation of strain rate of concrete under compression loading mode is lower than concrete under uniaxial tensile loading. Besides, a critical tensile strength and compressive strength can be observed when concrete under tension and compression, respectively. When the loading strain rate is lower than the critical strain rate, critical strength behavior linearity with strain rate and the critical strength increases with the increase of loading strain rate. However, as the loading strain rate is over the critical strain rate, which is generally 10^0^/s, the increase rate of strength is higher than that of strain [12,13]. The groups of Lin [14,15] and Song [16,17] studied dynamic properties of concrete at multi-axial load and found that the dynamic performance of concrete was significantly affected by strain rate and the concrete strength gradually increases with the increase of loading strain rate. Under the confining pressure condition, the increased rate of concrete strength was lower than that at the uniaxial loading condition. Besides, based on the experimental results, a dynamic failure criterion was proposed to relate dynamic increase rate with loading strain rate. For the research of SCC, many scholars mainly focus on the mix proportion, workability, durability and mechanical properties of SCC [18,19,20,21,22,23,24,25]. Specifically, mechanical properties under loading conditions including the uniaxial compression, uniaxial tension, bending, and multi-axial loading were studied. The compressive strength and tensile strength of SCC are basically the same as that of ordinary concrete under the same water–cement ratio. For the same strength grade, the elastic modulus of SCC is basically similar to that of ordinary concrete, while the strength of SCC shows better homogeneity and less dispersion [26]. In practice, SCC not only bears the static load, but also bears dynamic loads, such as earthquake, wind, impact and explosion, etc, under which circumstances the SCC exhibits obvious strain rate effect [27,28], i.e., the mechanical properties of SCC are influcenced by the strain rate. Therefore, the research of concrete dynamic performance has extremely value of civil engineering [4,5,29]. However, almost all the previous studies on the dynamic properties of concrete are focused on ordinary concrete. Few studies have been conducted on the dynamic performance of SCC. Therefore, it is keenly desired to study the dynamic properties of SCC.

In this study, to explore the dynamic performance of SCC, the compression tests at different loading rates were performed on ordinary concrete and SCC. Eight different strain rates (10^−5^–10^−1^/s) were selected for the compression tests. Based on the tests, the stress–strain curves and failure patterns of both types of concrete at different loading strain rate can be obtained. To quantify the dynamic characteristics of concrete, the peak stress, elastic modulus, and peak strain (strain corresponding to the peak stress) were extracted from the stress–strain curve to develop the correlation of loading strain rate with peak stress and elastic modulus. Moreover, a constitutive model based on the dynamic elastic–plastic damage theory was developed to predict the stress–strain curve of ordinary concrete and SCC.

## 2. Experimental Scheme

### 2.1. Specimen Preparation

In this study, the design strength for ordinary concrete and SCC is 30 MPa (C30 or SCC30). To prepare the ordinary concrete, ordinary Portland 42.5 cement, slag, fine aggregate (natural river sand), city tap water, crushed stone coarse aggregate with a diameter range of 5–16 mm were used. For preparing SCC, two more additives of slag and superplasticizer were used. The mix proportion of C30 was calculated with trial matching, adjustment and determination according to the design specification [30]. According to technical specification for self-compacting concrete application, the SCC30 was designed and carried out the initial mix proportion using the absolute volume method, then adjusted and determined [31]. The mixture designs for both concretes are shown in Table 1.

**Figure 1:**
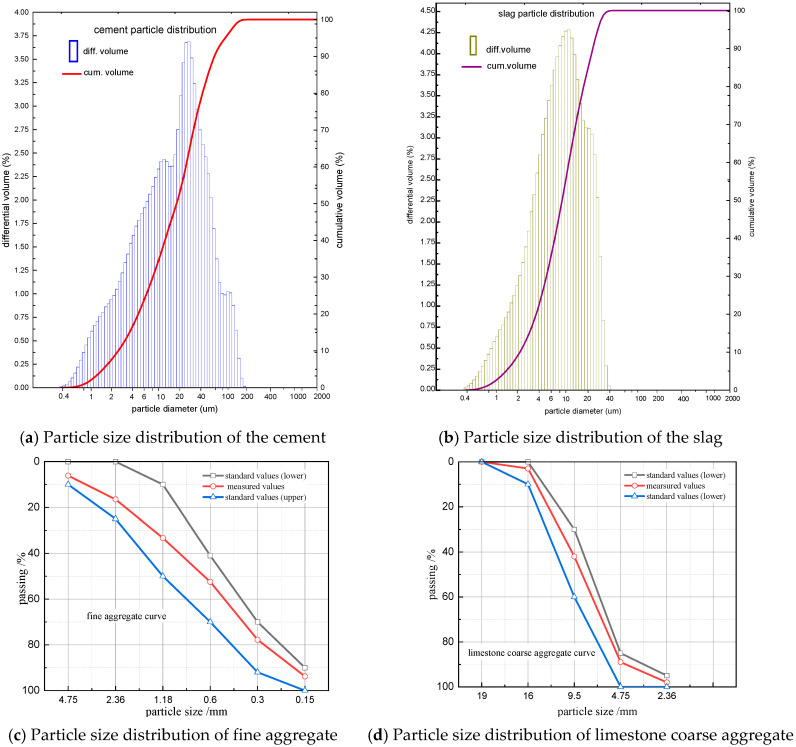
Physical properties of raw materials.

The chemical compositions of cement and slag used are shown in Table 2. The particle size distribution of them are shown in Figure 1a,b. The particle size distributions of fine aggregate (natural sand) and coarse aggregate (limestone) are shown in Figure 1c,d, respectively. The standard polycarboxylates high-performance water-reducer (Superplasticizer) is used. With the 1.0–2.0% superplasticizer, the water content was reduced by 20–30% while the concrete became SCC, the slump maintained, strength increased, shrinkage decreased, and environmental protection was achieved.

The casting methods for ordinary concrete and SCC are entirely different. For the ordinary concrete, according to the mix proportion in Table 1, first the coarse aggregate, fine aggregate, and cement were poured into the mixer for mixing, then the water was slowly poured into making them mix well, the mixed concrete was poured into the mold of 100 mm × 100 mm × 100 mm, and finally, the mold was placed on a vibrating table for compaction. For pouring of SCC, first, the cement, fine aggregate, and mineral powder were poured into the mixer for mixing, then the water with water reducing agent was poured into the mixer for mixing thoroughly, and finally, the coarse aggregate was poured into the mixer for mixing. After 24 h of curing, the molds were removed, and the ordinary concrete and SCC specimens were placed in a standard curing room for curing at 20 °C ± 3 °C and a humidity of 95% for 28 d, to develop strength [32].

### 2.2. Loading Program

**Figure 2:**
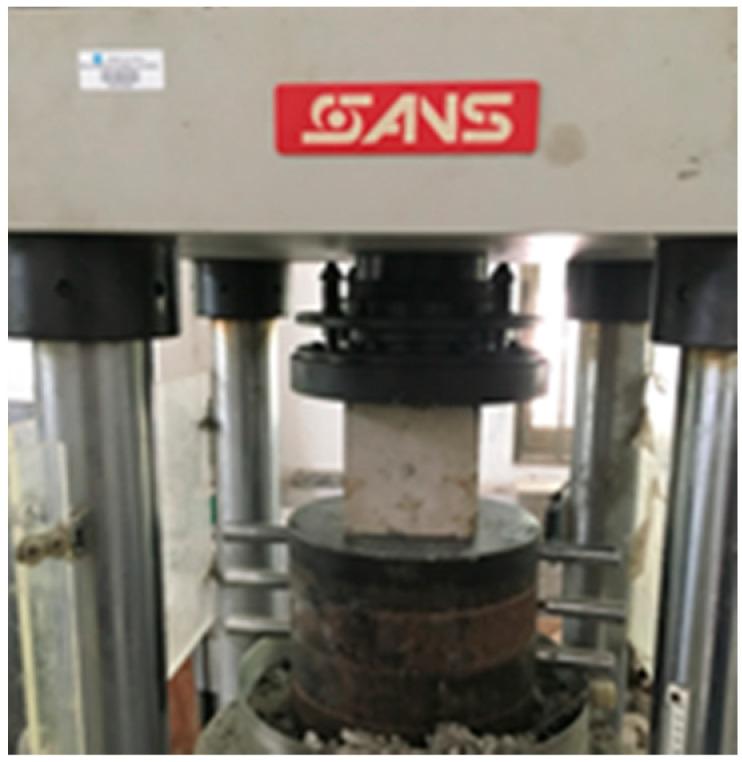
Experimental equipment.

In this paper, a hydraulic servo, as shown in Figure 1, was used to perform the test on the concrete specimens at different strain rates. This machine consists of a hydraulic actuating head, load-displacement acquisition system, and central control device. A high-precision load sensor and high-precision displacement sensor were equipped. During the test, data were automatically recorded by the supporting program of the testing machine. The load data collected by the force transducer can be calculated and converted to obtain the stress value, and the deformation data obtained by the displacement extensometer can be converted to obtain the strain data. Thus the analysis can meet the requirement loading and test results [9,10,33]. The accuracy meets the experimental requirements, as shown in Figure 2.

The cubic specimen with a size of 100 mm × 100 mm × 100 mm was adopted in this study. In the test, to eliminate the friction effect on the loading surface during the loading process, three layers of polyethylene plastic film and two layers of mechanical butter were applied on top and bottom contact surfaces of the concrete specimen. This friction reduction method has been highly recognized [10].

Concrete performs differently at different loading rates. Based on the loading rate from low to high, the actions of loads can be defined as creep (10^−8^–10^−6^*/*s), static load (10^−6^–10^−5^*/*s), seismic action (10^−3^–10^−2^*/*s), impact (1–10^2^*/*s), and explosion (10^2^–10^3^*/*s) [11]. Eight different loading strain rates were set, including 1.0 × 10^−5^/s, 5.0 × 10^−5^/s, 1.0 × 10^−4^/s, 5.0 × 10^−4^/s, 1.0 × 10^−3^/s, 5.0 × 10^−3^/s, 1.0 × 10^−2^/s, and 5.0 × 10^−2^/s. Among them, the loading rate of 1.0 × 10^−5^/s corresponds to a static load. Considering the magnitude of seismic action, the strain rate in the range from 1.0 × 10^−4^/s to 1.0 × 10^−1^/s is defined as a dynamic load. Three replicates were measured for each strain rate.

To conduct the compression test, a hybrid control loading mode coupling force and displacement controls were adopted. In the beginning, force control was used to provide a pre-pressing. The load was applied from 0 MPa to 0.05 *f_c_* and then unloaded to 0 MPa, which was repeated three times. After that, displacement control was used to apply the corresponding strain rate on the specimen until the specimen failed.

## 3. Results and Analysis

### 3.1. Failure Pattern

**Figure 3:**
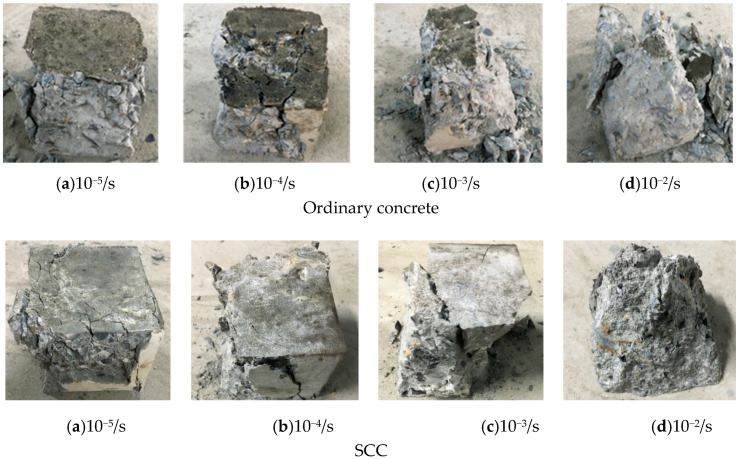
Failure pattern of concrete under compression with different loading strain rates.

From the failure pattern of concrete, the influence of loading strain rates on the mechanical properties of concrete can be identified. Figure 3 displays the failure patterns of ordinary concrete and SCC at different loading strain rates.

According to Figure 3, it can be observed that the cracks of ordinary concrete under compression with static loading strain rate and low dynamic loading strain rate are uniformly distributed on the non-loading surface. The crack development is relatively slow, then fewer cracks appeared, and the length of the crack development is relatively small. The failure of the specimen is mainly due to the failure of the cement gelling layer. However, at high strain rate load, the cracks developed rapidly, two main cracks were formed on the non-loading surface on the ordinary concrete specimen due to compression failure, it is accompanied by more smaller cracks, followed by more concrete pieces spalling, and the specimen cannot maintain its integrity. Although the influence of the loading strain rate on SCC is similar to that on ordinary concrete, SCC shows more brittle since the failure of concrete is dominated by diagonal crack. The failure of the two kinds of concrete can be attributed to the vertical pressure and the tensile strain on the non-loading surface under the influence of Poisson’s ratio. The specimen failed when the tensile strain reached its limit value. The reason why the two kinds of concrete have different static and dynamic failure is because under the action of static loading strain rate, the stress in the specimen has enough time to reach a uniform stress distribution. Thus, the concrete crack can be fully developed. However, with increasing loading strain rate, the stress in the specimen cannot reach a uniform distribution state in a very short time, and thus the crack cannot fully be developed. The difference in stress distributions at low and high loading strain rates leads to a significant difference in the failure patterns for both types of concrete.

**Figure 4:**
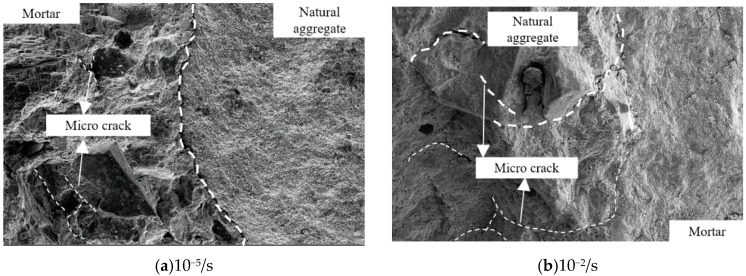
Microscopic failure pattern of self-compacting concrete (SCC) under different loading strain rates.

Further, SEM was used to analyze the pattern of SCC between the coarse aggregate and mortar gelling layer after compression failure under two different loading strain rates. Figure 4 shows the microscopic failure pattern.

According to Figure 4, at the loading strain rate of 10^−5^/s, the failure of SCC is mainly caused by the formation of cracks between the coarse aggregate and mortar gelling layer. When the loading strain rate increases to 10^−2^/s, the failure of concrete is not only caused by the apparent crack pattern between the coarse aggregate and mortar gelling layer but also caused by the apparent crack trace on some coarse aggregate, indicating that the failure of coarse aggregate occurred under high strain rate. The primary mechanism is that the loading time of the specimen is short, the uniform stress distribution cannot be formed inside the specimen, and the cracks cannot be fully developed, which leads to the crush of some coarse aggregates. As the coarse aggregate strength is higher than that between the coarse aggregate and mortar gelling layer, the mechanical properties of concrete under the action of high strain rate are different to some extent.

### 3.2. Stress–Strain Curve

**Figure 5:**
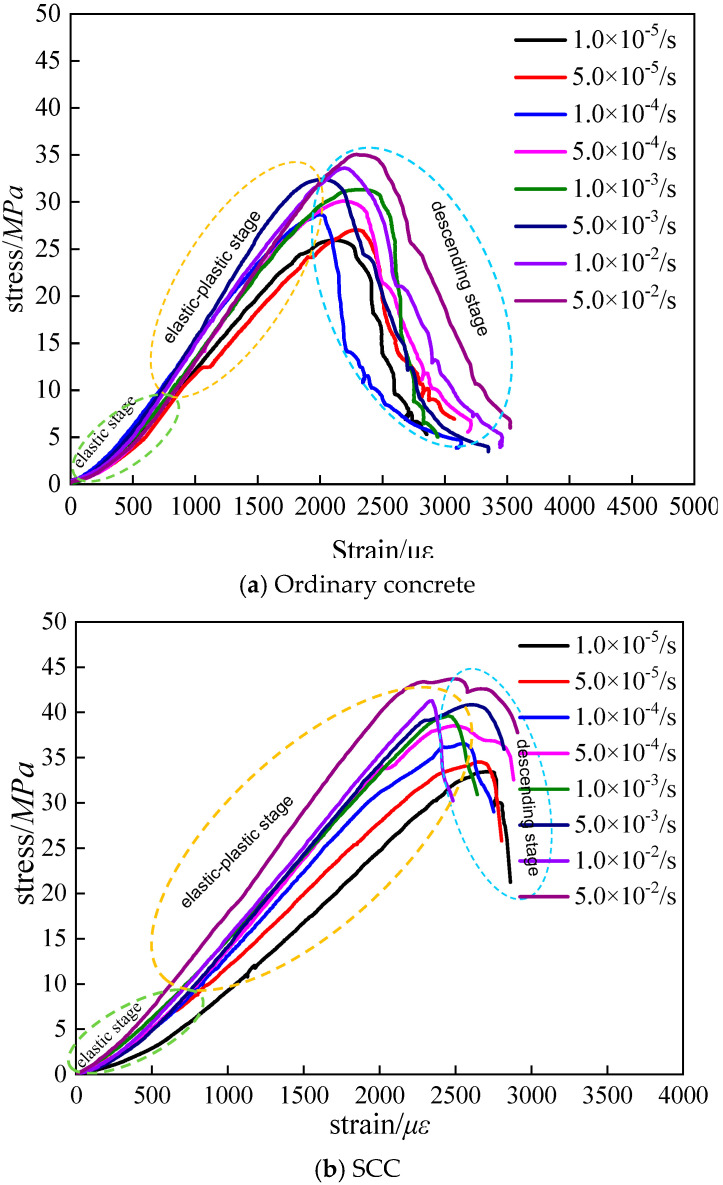
Stress–strain curve of concrete under uniaxial compression with different loading strain rates.

Based on the compression test for the ordinary concrete and SCC, the average value was calculated as the maximum strength, which did not meet, then the test was carried out again until the requirements were met [32]. In this study, 30 ordinary concrete specimens for eight different strain rates were tested, among which, one repeated test was conducted because the test result did not meet for the strain rate of 5 × 10^−3^/s and 5 × 10^−2^/s, and 27 SCC specimens were tested, including a repeated test for the strain rate of 1 × 10^−2^/s. Each stress–strain curve was plotted by the average value of three replicates. According to test data processing and analysis, the deviation of maximum strength of ordinary concrete and SCC for each strain rate is 0.8~1.3 and 0.7~1.4, respectively. Stress–strain curves of ordinary concrete and SCC with eight different loading strain rates were obtained. By analyzing the stress–strain curves of the two kinds of concrete, relevant mechanical properties can be revealed, shown in Figure 5. Meanwhile, the stress–strain curve also serves as an essential basis for analyzing other mechanical properties. Figure 5 presents the stress–strain curves of ordinary concrete and SCC. It is noted that the ordinate *y* represents the strength value of ordinary concrete or SCC, and the coordinate x represents the engineering strain value ε in Figure 5. The so-called engineering strain is relative to the true strain *ε*_t_. Engineering strain is defined as the elongation ∆*l* within the range of gauge length divided by the initial value *l*_0_ of the gauge length, written as ε = ∆*l*/*l*_0_. In the process of calculating real strain value, usually, the whole stretching stage is divided into countless tiny segments. for any segment, the instantaneous length of the specimen is *l*, the elongation of the specimen is *dl*, the change of the true strain is *dε*_t_ = *dl*/*l*, the true strain of the specimen stretching from *l*_0_ to *l* can be regarded as the cumulative value of infinitely many true strain increments, and the equation is written as *ε*_t_ = $\int^{\;}d\varepsilon_{t}=\int_{l_{0}}^{l}\frac{dl}{l}=ln\frac{l}{l_{0}}$.

According to the analysis of Figure 5, the development of stress–strain curves of ordinary concrete and SCC shows good continuity and smoothness at different loading strain rates, and the loading strain rate does not influence the continuity and smoothness of the concrete stress–strain curve. The stress–strain curves of ordinary concrete and SCC under different loading strain rates are divided into three phases: elastic stage, elastic–plastic stage, and descending stage. Besides, from Figure 5, it can also be observed that the peak compressive stress and dissipation energy gradually increase with the increase of loading strain rate. Compared with the stress–strain curve of ordinary concrete, the brittleness of SCC is obvious.

**Figure 6:**
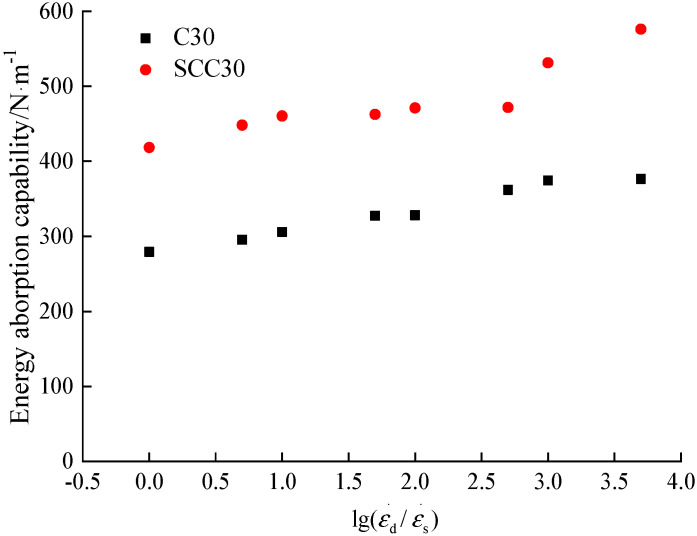
The effect of strain rate on energy absorption capacity.

**Figure 7:**
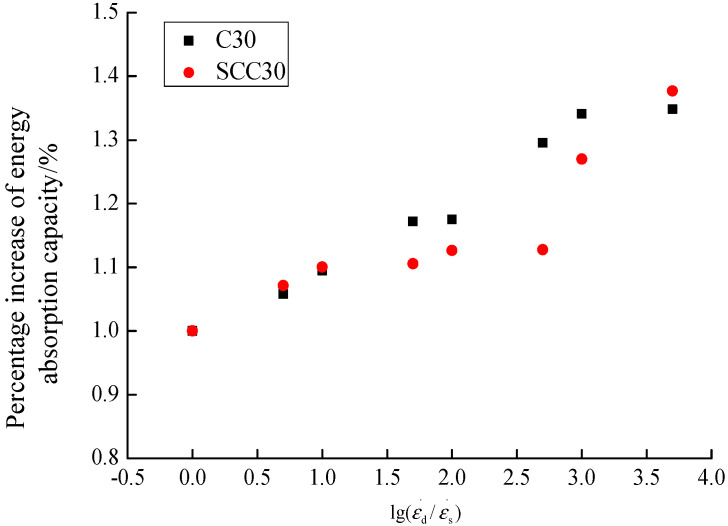
The influence of strain rate on the percentage increase of energy absorption capacity.

The energy absorption capacity of concrete is generally considered as the energy required from initial loading to failure of the specimen [14]. According to the stress–strain curves of ordinary concrete and SCC under different loading conditions, the influence of different loading strain rates on concrete energy absorption capacity is calculated, as shown in Figure 6 and Figure 7.

According to the analysis in Figure 6 and Figure 7, the energy absorption capacity of ordinary concrete and SCC gradually increases obviously with the increase of the loading strain rate. The energy absorption capacity of ordinary concrete increased from 279.32 N/m at strain rate 1.0 × 10^−5^/s to 376.63 N/m at strain rate 5.0 × 10^−2^/s, and the maximum percentage increase was 34.84%. The energy absorption capacity of SCC increased from 418.45 N/m at strain rate 1.0 × 10^−5^/s to 576.16 N/m at strain rate 5.0 × 10^−2^/s, with a maximum percentage increase of 37.69%. From the above analysis, it can be seen that the energy absorption capacity of SCC is higher than that of ordinary concrete in the study of this paper, and the energy absorption capacity of the two kinds of concrete is generally the same under the influence of loading strain rate.

### 3.3. Peak Stress

To study the influence of strain rate on the peak stress in the stress–strain curve, the dynamic increase coefficient is commonly used [9]. The formula can be expressed as follows:

(1) $$\alpha_{DIF}{=\sigma }_{d}{/\sigma }_{s}$$

where $\sigma_{s}$ is the peak compressive stress at the static strain rate of 1.0 × 10^−5^/s; $\sigma_{d}$ is the peak compressive stress at the dynamic strain rate. In this study, the seven strain rates in the range of 5.0 × 10^−5^/s–5.0 × 10^−2^/s were included.

**Figure 8:**
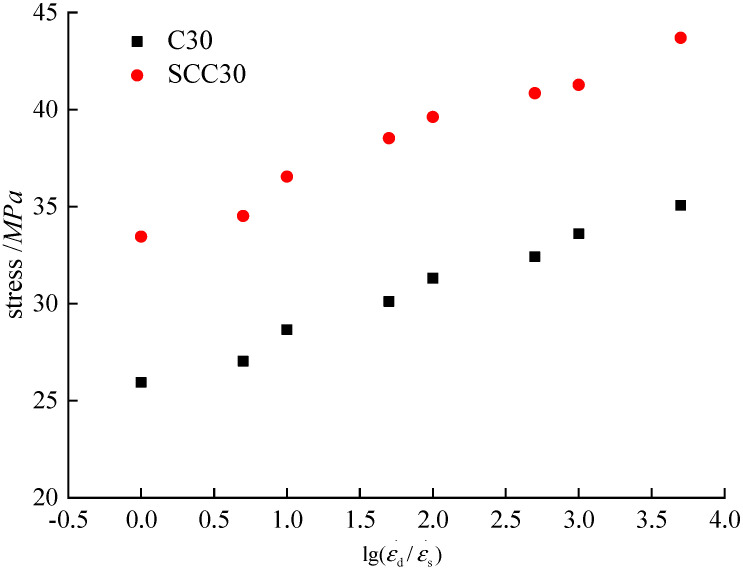
Relationship between strain rate and peak compressive stress.

According to the stress–strain curves of ordinary concrete and SCC, the peak stresses of the two kinds of concrete at different loading strain rates can be extracted. By inputting into Formula (1), the dynamic increase coefficient can be obtained. Figure 8 presents the peak compressive stresses and corresponding dynamic increase coefficients of ordinary concrete and SCC at different loading strain rates.

According to Figure 8, the peak stress of ordinary concrete and SCC increase with the increase of loading strain rate. When the loading strain rate is 1.0 × 10^−5^/s, the peak compressive stress of ordinary concrete and SCC reach 25.94 MPa and 33.45 MPa, respectively. When the loading strain rate increases to 5.0 × 10^−2^/s, the peak compressive stress of ordinary concrete and SCC are 35.07 MPa and 43.69 MPa, respectively. The increases of peak stress are 35% and 31% for ordinary concrete and SCC, respectively. According to Sparks [6] and Bischoff’s [7] experimental studies, when the maximum strain rate is 5.0 × 10^−2^/s, the peak compressive stress of concrete increases by about 40% compared with that under static loading condition; Song [10,11] selected the loading strain rate of 2.0 × 10^−5^/s–2.0 × 10^−2^/s for an experimental study of the ordinary concrete under uniaxial compression, and the peak compressive stress increased by 34.26%; the results of Li Jie’s [8,9] experimental study on ordinary concrete under uniaxial compression with different strength grades showed that considering the loading strain rate of 1.0 × 10^−5^/s to 1 × 10^−1^/s, the peak compressive stress generally increased to a range of 30%–40%. Thus, the increase of the peak stress of ordinary concrete in this study is consistent with the findings in the above references while the increase of the peak stress of SCC is slightly lower than that of ordinary concrete. The peak compressive stress of the two kinds of concrete increased gradually with the increase of loading strain rates, which can be ascribed to the hysteretic effect. Due to the delay of concrete damage at high strain rate, the peak compressive stress of concrete at the static loading strain rate is lower than concrete at the dynamic loading strain rate.

In order to further quantitatively analyze the influence of loading strain rate on peak compressive stress of ordinary concrete and SCC, a dimensionless dynamic increase coefficient was established by taking the logarithm of the dynamic strain rate and static strain rate [34]. The dimensionless dynamic increase coefficient can be expressed as follows:

(2) $$\alpha_{DIF}=1+a\mathrm{lg}(\varepsilon_{d}/\varepsilon_{s})$$

**Figure 9:**
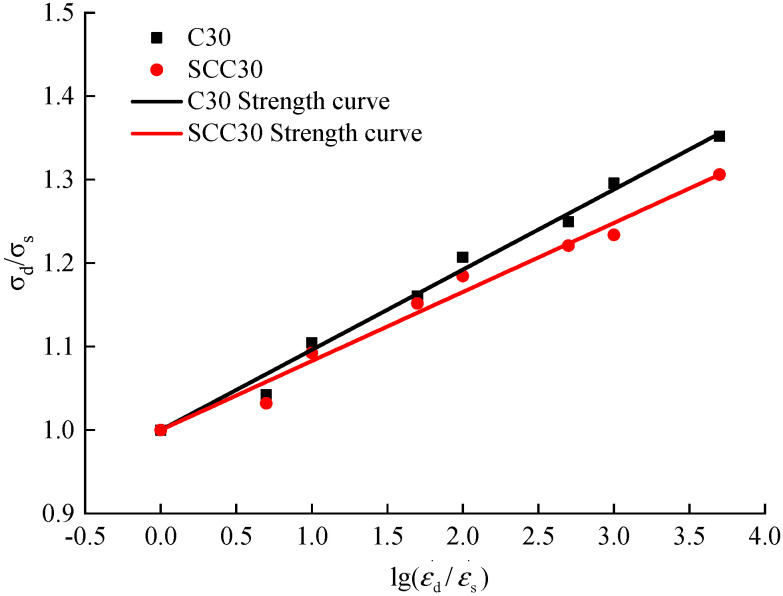
Relationship between strain rate and dynamic increase coefficient of peak stress.

According to the experimental data of peak stress of ordinary concrete and SCC at different loading strain rates in this paper, the mathematical regression analysis was carried out by using Formula (2) to obtain the relation equation for dynamic increase coefficient and loading strain rate of ordinary concrete and SCC, as shown in Figure 9 and Formulas (3) and (4).

(3) $$\mathrm{Ordinary}\;\mathrm{concrete}:\;\alpha_{\mathrm{DIF}}=1+0.09608\mathrm{lg}\left(\varepsilon_{d}/\varepsilon_{s}\right)\;R^{2}=0.9998$$

(4) $$\mathrm{SCC}:\;\alpha_{\mathrm{DIF}}=1+0.08271\mathrm{lg}\left(\varepsilon_{d}/\varepsilon_{s}\right)\;R^{2}=0.9997$$

According to Figure 9 and Formulas (3) and (4), it can be found that with the increase of loading strain rate, the peak compressive stresses of ordinary concrete and SCC increase obviously. Besides, the dimensionless log of the dynamic increase coefficient of peak compressive stress shows linearly with the increase of loading strain rate. Thus the proposed Formula (2) can better quantify such variation. According to the slope in Formulas (3) and (4), the slope in the equation of SCC is slightly lower than that of ordinary concrete, which further demonstrates that the peak compressive stress of SCC under the influence of loading strain rate is lower than that of ordinary concrete.

### 3.4. Elastic Modulus

Elastic modulus is one of the essential parameters to characterize the mechanical properties of concrete. In this paper, according to the compressive stress–strain curves of ordinary concrete and SCC in Figure 5, the elastic moduli of concrete under different loading conditions were derived from studying the influence of loading strain on the elastic modulus of concrete. The elastic modulus can be calculated by the following equation:

(5) $$E=\frac{\sigma_{0.5}-\sigma_{0.2}}{\varepsilon_{0.5}-\varepsilon_{0.2}}$$

where $\sigma_{0.5}$ and $\sigma_{0.2}$ are respectively 50% and 20% of the peak stress of concrete, $\varepsilon_{0.5}$ and $\varepsilon_{0.2}$ are respectively 50% and 20% of the peak strain of concrete.

**Figure 10:**
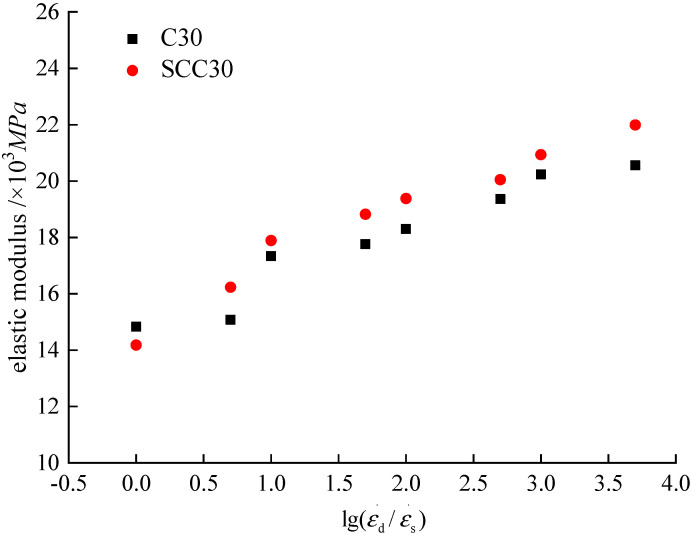
Relationship between strain rate and elastic modulus.

According to the stress–strain curves under different loading strain rates in this paper, the elastic modulus of the two kinds of concrete can be obtained by using Formula (5). Additionally, a similar Formula (1) was used to obtain the dynamic increase coefficient of elastic modulus of concrete under different loading conditions, as shown in Table 3 and Figure 10.

According to Table 3 and Figure 10, we can find that with the increase of loading strain rate, the elastic modulus of ordinary concrete and SCC gradually increase. When the loading strain rate is 1.0 × 10^−5^/s, the elastic modulus of ordinary concrete and SCC are 14.83 × 10^3^ MPa and 14.18 × 10^3^ MPa, respectively. When the loading strain rate is 5.0 × 10^−2^, the elastic modulus of ordinary concrete and SCC are 20.56 × 10^3^ MPa and 21.99 × 10^3^ MPa, which increases by 36% and 48%, respectively. In this literature [9,17,18], the strain rate was considered within the range of 1 × 10^−5^/s~5 × 10^−2^/s. The compressive dynamic performance of ordinary concrete was studied. The elastic modulus of ordinary concrete was increased by 35%~45% under the influence of strain rate, which was basically consistent with the conclusions of this paper. In contrast, the influence of the loading strain rate on elastic modulus of SCC is higher than that of ordinary concrete.

The same quantitative analysis method as the peak stress was used to analyze the variation of the elastic modulus of ordinary concrete and SCC under the influence of loading strain rate, and the relation equation for the increase coefficient of elastic modulus of concrete and the loading strain rate was established, as shown in Formula (6).

(6) EdEs=1+blg(εdεs)

According to the elastic modulus of ordinary concrete and SCC with different loading strain rates in this paper, mathematical regression analysis was carried out by using Formula (6), and the relationship between the increase coefficient of elastic modulus of the two kinds of concrete and the loading strain rate was obtained, as shown in Figure 7 and Formulas (7) and (8).

**Figure 11:**
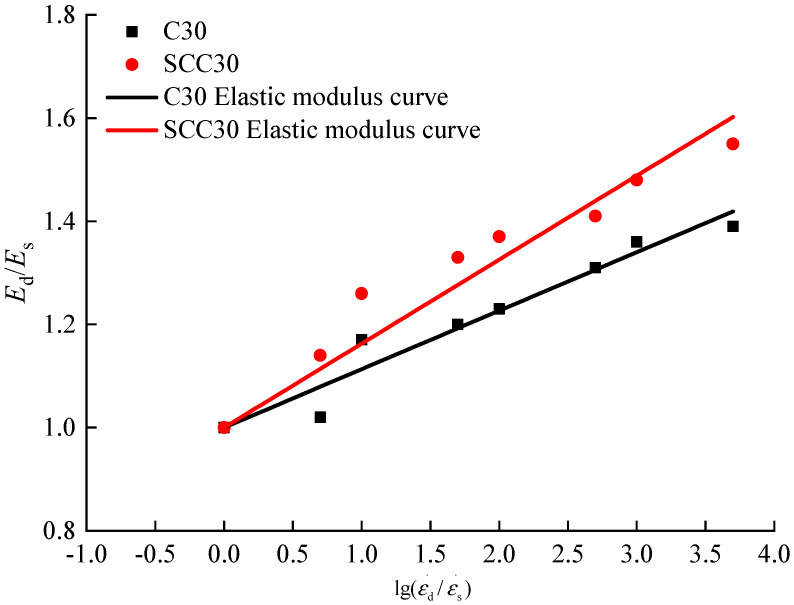
Relationship between strain rate and dynamic increase coefficient of elastic modulus.

According to Figure 11 and Formulas (7) and (8), it can be observed that with the increase of loading strain rate, the elastic moduli of ordinary concrete and SCC increase gradually. Besides, the dimensionless log of the dynamic increase coefficient of elastic modulus and the loading strain rate varies linearly, and the proposed Formula (6) could better describe the relationship between dynamic increase coefficient of elastic modulus and loading strain rate. According to the comparative analysis of the slope in Formulas (7) and (8), the elastic modulus of ordinary concrete under the influence of loading strain rate is lower than that of SCC, which is consistent with the conclusion of qualitative analysis.

(7) Ordinary concrete: EdEs=1+0.11327lg(εdεs) R2 = 0.9992

(8) SCC: EdEs=1+0.162731lg(εdεs) R2 = 0.9985

### 3.5. Peak Strain

**Figure 12:**
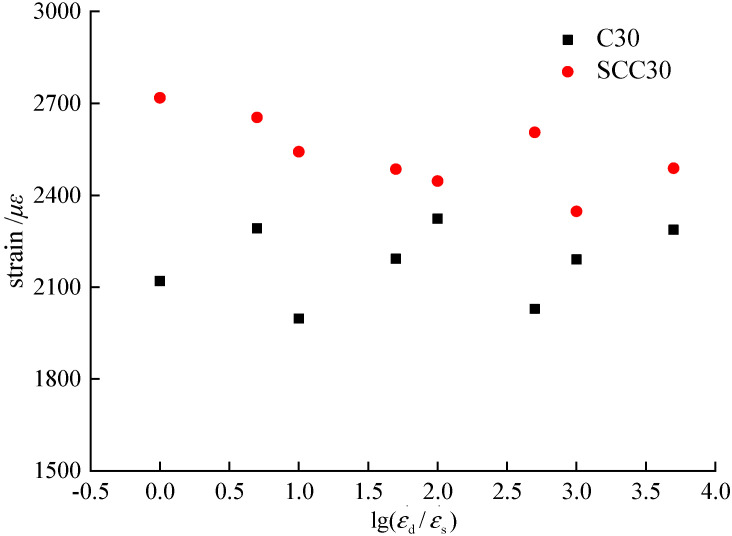
Relationship between strain rate and peak compressive strain.

**Figure 13:**
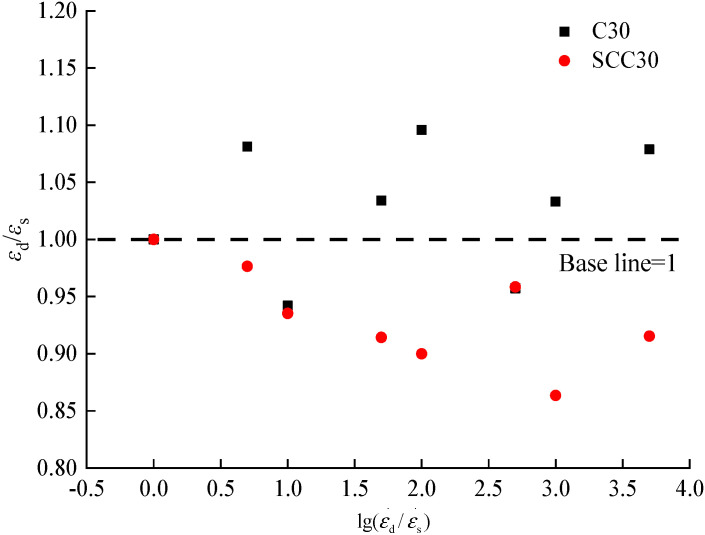
Relationship between strain rate and variation factor of peak strain.

In order to analyze the effect of loading strain rate on the peak strain, an analysis method similar to that of the peak compressive stress was used. The peak strain rate was extracted from the stress–strain curve in Figure 4 to obtain the relationship between loading strain rate and peak strain and the relationship between loading strain rate and variation factor of peak strain, as shown in Figure 12 and Figure 13.

According to Figure 12 and Figure 13, the peak compressive strains of ordinary concrete and SCC at different loading strain rates are in the ranges of 1997 *με-*2323 *με* and 2347 *με*-2718 *με*, respectively. The peak compressive strain rate of SCC is higher than that of ordinary concrete. The peak compressive strain rate of ordinary concrete under loading strain rate of 1.0 × 10^−5^–5.0 × 10^−2^ is -5.80–+9.58%, while that of SCC is −13.65–0%. With the increase of loading strain rate, the variation of the peak compressive strains is discrete without a clear pattern, while the peak strain of SCC decreases gradually with the increase of loading strain rate, which can be ascribed to the randomness and rate-dependent coupling effect of concrete, resulting in the discrete variation of peak strain.

The effect of loading strain rate on the peak compressive strain of ordinary concrete can be summarized as follows: (1) with the increase of loading strain rate, the peak compressive strain of concrete would gradually increase; (2) with the increase of loading strain rate, the peak compressive strain of concrete would gradually decrease; (3) the peak compressive strain of concrete was not influenced by the loading strain rate. We found that the performance of ordinary concrete is consistent with the third point, while SCC is consistent with the second point. This difference is caused by the coupling effect of the randomness and discreteness of concrete and loading strain rate [9].

## 4. Constitutive Theory of Dynamic Elastic–Plastic Damage

### 4.1. Constitutive Theory of Elastic–Plastic Damage

The basic theory of the energy-based constitutive model of static elastic–plastic damage is the effective stress tensor in the continuum damage mechanics [8,33].

(9) $$\bar{\sigma }=E_{0}:\varepsilon^{e}=E_{0}:(\varepsilon -\varepsilon^{p})={\bar{\sigma }}^{+}+{\bar{\sigma }}^{-}=E^{+}:\bar{\sigma }+E^{-}:\bar{\sigma }$$

where $E_{0}\;$ is the initial stiffness tensor of concrete; ε $\varepsilon^{e}$ and $\varepsilon^{p}$ are respectively the total strain tensor, elastic strain tensor and plastic strain tensor; $E^{+}$ and $E^{-}$ are respectively positive and negative projection tensor of $\bar{\sigma }$.

The damage evolution of concrete is irreversible, leading to the reduction of concrete’s energy dissipation. Damage variable $d^{-}$ can be used to describe the influence of concrete shear damage on the reduction of elastic strain energy of concrete materials concerning concrete compression failure. According to the basic theory of continuum damage mechanics, the constitutive equation of elastic–plastic damage of concrete can be obtained:

(10) $$\sigma =(1-d^{-}){\bar{\sigma }}^{-}=\left(1-D\right):\bar{\sigma }$$

where I is fourth-order consistency tensor; D is fourth-order damage tensor, which can be expressed as:

(11) $$D=d^{-}E^{-}$$

The evolution law of $\varepsilon^{p}$ plastic strain can be determined by the spatial plastic mechanics of effective stress. It can be simplified as follows:

(12) $$\varepsilon^{p}=b^{p}\bar{\sigma }$$

(13) $$b^{p}=E\xi^{p-}H\left(d^{-}\right)\frac{\langle \varepsilon^{e}:\varepsilon \rangle }{\bar{\sigma }:\bar{\sigma }}\geq 0$$

where E is the initial elastic modulus of concrete; *H*(_o_) and 〈o〉 are respectively Heaviside function and McAuley function; $\varepsilon^{p-}\geq 0$ indicates model parameter of plastic flow in concrete under compression.

According to relevant references [8], the plastic Helmholtz free energy potential of concrete was derived, and the elastic–plastic damage energy release rate was obtained:

(14) $$Y^{-}=\alpha \bar{I_{1}}+\sqrt{3\bar{J_{2}}}$$

(15) α=fccfc−12fccfc−1

where $\bar{I_{1}}$ and $\bar{J_{2}}$ are respectively the first invariant of effective stress tensor $\bar{\sigma }$ and the second invariant of deviator; α is a concrete parameter, $f_{cc}$ is the ultimate strength of concrete under biaxial constant pressure, and $f_{c}$ is the ultimate strength of concrete under uniaxial compression. In this paper, fccfc of ordinary concrete and SCC is generally 1.16, so α is 0.1212.

According to the formula above, the threshold value $r_{0}{}^{-}$ of the initial elastic–plastic damage energy release rate of concrete is:

(16) $$r_{0}{}^{-}=\left(1-\alpha \right)f_{0}$$

where $f_{0}$ is the linear-elastic ultimate strength of concrete under uniaxial compression, generally 30–60% of uniaxial compressive strength of concrete.

The state equation $G^{-}$ of damage variable $d^{-}$ can thus be obtained, i.e., damage criterion:

(17) $$G^{-}\left(Y_{n}{}^{-},r_{n}{}^{-}\right)=Y_{n}{}^{-}-r_{n}{}^{-}\leq 0$$

where the subscript *n* is the current time; $Y_{n}{}^{-}$ is the elastic–plastic damage energy release rate at the current time; $r_{n}{}^{-}$ is the threshold value of elastic–plastic damage energy release rate of concrete at the time. If $G^{-}\left(Y_{n}{}^{-},r_{n}{}^{-}\right)\leq 0$, i.e., $d^{-}=0$, the concrete is at the stage of damage unloading or neutral load varying, and the damage will not be further developed, if $G^{-}\left(Y_{n}{}^{-},r_{n}{}^{-}\right)=0$, i.e., $d^{-}\geq 0$, the concrete is in a state of damage loading.

According to the above damage criterion, the damage variable is expressed as follows:

(18) $$d^{-}=1-\frac{r_{0}{}^{-}}{r_{n}{}^{-}}\left(1-A^{-}\right)-A^{-}\exp\left[B^{-}\left(1-\frac{r_{0}{}^{-}}{r_{n}{}^{-}}\right)\right]$$

where $A^{-}$ and $B^{-}$ are model parameters, which can be calibrated by the stress–strain curve of concrete under uniaxial compression, i.e., 1.0 and 0.213 in this paper. Once the evolutional rule of the threshold value $r^{-}$ of damage energy release rate is determined, the constitutive relation of elastic–plastic damage of concrete can be obtained.

It is necessary to improve the strain rate dependent damaged material of concrete so that it can describe the influence of strain rate effect such as the increase of dynamic strength and the decrease of material nonlinearity. The influence of strain rate on damage state and evolution of concrete was considered by regularizing the viscosity of the threshold value $r^{-}$ of damage energy release rate, thus obtaining:

(19) $$r^{-}=\mu^{-}\phi^{-}\left(G^{-}\right)=\mu^{-}\langle G^{-}/r^{-}\rangle {}^{a^{-}}=\mu^{-}\langle Y^{-}/r^{-}-{1\rangle }^{a^{-}}$$

where $\phi^{-}$ is the flow function of the threshold value of viscous damage energy release rate; $\mu^{-}$ is the flow constant of the threshold value of damage energy release rate; $a^{-}$ is concrete material constant.

If the flow constant $\mu^{-}$ of the threshold value of damage energy release rate approaches 0, i.e., $r^{-}$ approaches 0, $d^{-}$ will approach 0; if the flow constant $\mu^{-}$ of the threshold value of damage energy release rate approaches ∞, i.e., 〈ϕ(G)〉=r/μ approaches 0, r will approach Y and $\dot{r}$ will approach $\dot{Y}$.

The evolutional rule of the threshold value of damage energy release rate can be obtained from the above formulas, and according to the constitutive relation of elastic–plastic damage and the evolutional rule of damage, the complete constitutive model of elastic–plastic damage of concrete considering strain rate effect can be obtained.

### 4.2. Dynamic Expansion of Damage Energy Release Rate

For concrete strain rate-related damage materials, inspired by Perzyna visco-plastic regularization of plastic flow constant in visco-plastic flow rule, the influence of strain rate on damage state and evolution of materials can be considered by regularizing the viscosity of the threshold value $r^{-}$ of damage energy release rate. In this study, the evolutional rule of the threshold value $r^{-}$ of the damage energy release rate is as follows:

(20) $$r^{-}=\mu^{-}\phi^{-}\left(G^{-}\right)$$

(21) $$\phi^{-}\left(G^{-}\right)=\langle G^{-}/r^{-}\rangle {}^{a^{-}}=\langle G^{-}/r^{-}-{1\rangle }^{a^{-}}$$

where $\phi^{-}$ is the flow function of the threshold value of viscous damage energy release rate; $\mu^{-}$ is the flow constant of the threshold value of damage energy release rate; $a^{-}$ is material constant. According to an experimental study on concrete under uniaxial compression with different loading strain rates, it was determined that $\mu^{-}$= 6.0 × 10^10^ N/s, $a^{-}$= 4.5.

According to Formula (25), if the flow constant $\mu^{-}$ of the threshold value of damage energy release rate approaches the limit value, the following can be obtained:

(22) $$\{\begin{array}{c} \mu^{-}\to 0\Rightarrow {\dot{r}}^{-}\to 0\Rightarrow {\dot{d}}^{-}\to 0\;\;\;\;\;\;\;\;\;\;\;\;\;\;\;\;\\ \mu^{-}\to \infty \Rightarrow \langle \phi^{-}(G^{-})\rangle ={\dot{r}}^{-}/\mu^{-}\to 0\Rightarrow \{\begin{array}{c} r^{-}\to Y^{-}\\ {\dot{r}}^{-}\to {\dot{Y}}^{-} \end{array} \end{array}$$

Therefore, Perzyna regularization of the threshold value of damage energy release rate includes the general form of damage threshold evolution, including the elastic–plastic constitutive model and the constitutive model of elastic–plastic damage related to strain rate.

After the evolutional rule of the threshold value of damage energy release rate determined above, the constitutive model of elastic–plastic damage of concrete with strain rate effect considered can be formed according to the constitutive model of elastic–plastic damage of concrete in Section 3.1.

### 4.3. Experimental Verification

**Figure 14:**
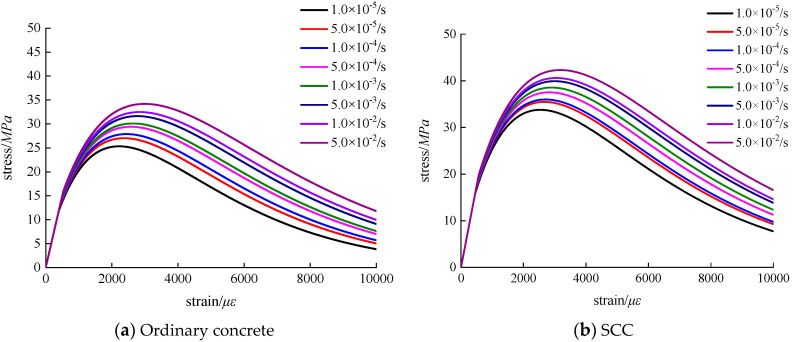
Calculated constitutive relation of elastic–plastic damage of concrete.

**Figure 15:**
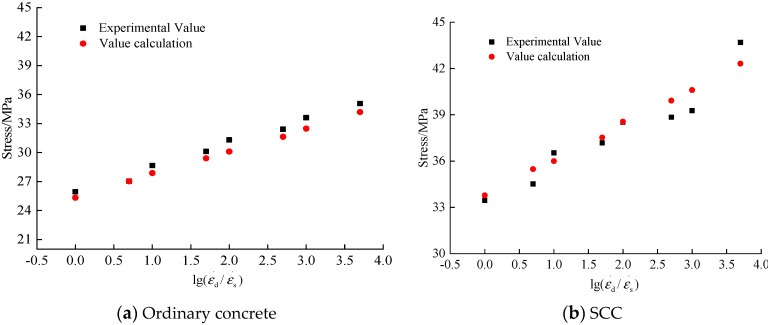
Calculated and theoretical peak compressive stress of concrete.

To verify the applicability of the proposed constitutive model, numerical analysis was conducted to simulate the ordinary concrete and SCC under compression at different strain rates. The peak stress of concrete under uniaxial compression, elastic modulus, and Poisson’s ratio were determined based on the experimental data of ordinary concrete and SCC in this paper. The Poisson’s ratio of ordinary concrete is 0.20 and that of SCC is 0.22. The predicted stress–strain curves of ordinary concrete and SCC based on the constitutive model are plotted in Figure 14 and Figure 15, respectively.

According to Figure 14 and Figure 15, the constitutive model of dynamic elastic–plastic damage of concrete proposed in this paper could better describe the strain rate effect of ordinary concrete and SCC. With the increase of strain rate, the peak stress of ordinary concrete and SCC both increased obviously. According to Figure 15, the peak stresses predicted by the constitutive model of dynamic elastic–plastic damage are in good agreement with the peak stresses obtained from tests.

## 5. Conclusions

This study experimentally investigated the dynamic properties of SCC under compression at different loading strain rates. The following conclusions can be drawn:

(1) SCC shows a similar compression failure pattern to ordinary concrete. From the low static loading strain rate to the high dynamic loading strain, the failure of concrete converts from due to the mortar and the coarse aggregate gelling surface to the failure coarse aggregate. The failure of SCC shows obvious brittleness at high loading strain rate compared with ordinary concrete.

(2) With the increase of loading strain rate, both the peak compressive stress and elastic modulus of ordinary concrete and SCC increases gradually. The peak compressive strain of the two kinds of concrete under the influence of strain rate is discrete, and the dimensionless log of the dynamic increase coefficient of peak compressive stress and elastic modulus and the loading strain rate varies linearly.

(3) The increase of peak compressive stress of ordinary concrete under the influence of the loading strain rate is slightly higher than that of SCC.

(4) In this paper, based on the theory of elastic–plastic damage, the dynamic constitutive model of ordinary concrete and SCC was established through the dynamic expansion of damage energy release rate. According to a comparative analysis of the experimental data and calculated result, the proposed dynamic constitutive model is applicable to ordinary concrete and SCC.

## Figures and Tables

**Table 1:** Mix proportion of concrete.

Concrete Strength	Mass of Ingredients of Concrete Per Cubic Meter/Kg					
	Cement	Water	Coarse Aggregate	Fine Aggregate	Slag	Superplasticizer
C30	178	279	1034	780	-	-
SCC30	300	123	865	781	200	3.20

**Table 2:** Chemical composition of the cement and slag.

Types	Chemical Composition/%								
	SiO_2_	Al_2_O_3_	Fe_2_O_3_	MgO	CaO	SO_3_	R_2_O	MnO	H_2_O
Cement	20.80	5.53	3.89	1.70	62.31	2.62	0.52	0	0
Slag	23.35	13.42	14.03	7.56	37.46	0	0.61	0	0

**Table 3:** Elastic modulus of ordinary concrete and SCC (×10^3^ MPa).

Strain Rate	Ordinary Concrete		SCC	
	Elastic Modulus	Dynamic Increase Coefficient	Elastic Modulus	Dynamic Increase Coefficient
1.0 × 10^−5^	14.83	1.00	14.18	1.00
5.0 × 10^−5^	15.08	1.02	16.23	1.14
1.0 × 10^−4^	17.34	1.17	17.89	1.26
5.0 × 10^−4^	17.76	1.20	18.82	1.33
1.0 × 10^−3^	18.30	1.23	19.38	1.37
5.0 × 10^−3^	19.36	1.31	20.05	1.41
1.0 × 10^−2^	20.24	1.36	20.94	1.48
5.0 × 10^−2^	20.56	1.39	21.99	1.55
